# Microbial Diversity of the Red Sea Urchin Loxechinus albus during Controlled Farming in Puerto Montt, Chile, Using 16S rRNA Gene Amplicon Sequencing

**DOI:** 10.1128/MRA.00851-19

**Published:** 2019-10-17

**Authors:** Daniel A. Medina, Rudy Suárez, Marcos Godoy

**Affiliations:** a Laboratorio de Biotecnología Aplicada, Facultad de Medicina Veterinaria, Universidad San Sebastián, Puerto Montt, Chile; b Centro de Investigaciones Biológicas Aplicadas (CIBA), Puerto Montt, Chile; c Doctorado en Acuicultura, Programa Cooperativo Universidad de Chile, Universidad Católica del Norte, Pontificia Universidad Católica de Valparaíso, Valparaíso, Chile; Georgia Institute of Technology

## Abstract

Loxechinus albus is a shallow-water sea urchin, and its distribution is related to the cold water of the Southern Hemisphere. Recently, bacterial communities, also called microbiota, in sea urchins have started being explored. In this report, we have characterized the surface, testa, and gonad microbiota using 16S rRNA sequencing.

## ANNOUNCEMENT

Young specimens of Loxechinus albus (*n* = 11, less than 5 cm in diameter) were collected from Experimental Fish Farming of Aquaculture and Marine Sciences of Metri (C74W+7H Lenca, Puerto Montt, Chile). It should be noted that the sea urchins were grown in 5-m^2^ tanks using a closed recirculatory seawater system in a farming density of 70 specimens per m^2^ and fed using giant kelp algae (Macrocystis pyrifera). After collection, the red sea urchins were transported alive using a portable plastic cooler container (Coleman, USA) filled with seawater to a maximum density of three specimens per container. Sample processing was performed in the Laboratorio de Biotecnología Aplicada of Universidad San Sebastián, Puerto Montt, Chile, which is located 29 km away from the experimental station. Sterile swabs were used to recover microbial material from the surface, while 0.5 g of gonad and testa was collected by necropsy using sterile scalpels and tweezers. Furthermore, the samples were stored in RNAlater (Sigma, USA), and nucleic acid extractions were conducted using a modified protocol to recover microbial DNA based on phenol-chloroform extraction and glass bead-beating tissue homogenization (1). To facilitate bacterial wall rupture, a combination of lysozyme and proteinase K enzymatic digestion was used before bead beating homogenization (2). Additionally, DNA integrity was analyzed by 1% agarose gel electrophoresis, and nucleic acid quantification was performed using the NanoDrop lite device at 260 nm (Thermo Scientific, USA). A total of 500 ng of DNA was sent to the Molecular Research DNA LP laboratory (MR DNA, USA) for library preparation and DNA sequencing. The 16S rRNA gene V4 variable region was amplified using the 515F and 806R primers (3) that were previously used to characterize the microbiota of the sea urchin Lytechinus variegatus (4) and the purple sea urchin Strongylocentrotus purpuratus (5). PCR amplification was performed in a single-step 30-cycle PCR using the HotStarTaq plus master mix kit (Qiagen, USA) under the following conditions: 94°C for 3 minutes, followed by 30 cycles of 94°C for 30 seconds, 53°C for 40 seconds, and 72°C for 1 minute, after which a final elongation step at 72°C for 5 minutes was performed. After the amplification, the PCR products were first checked in a 2% agarose gel to determine the quality of amplification and then used to prepare a DNA library using the TruSeq DNA LT sample prep kit (Illumina, USA) following the manufacturer’s instructions. Sequencing was performed on a MiSeq instrument (Illumina, USA), obtaining a yield of 20,000 reads per sample, with a length of 2 × 250-bp paired-end reads. Raw data were downloaded from Illumina BaseSpace Sequence Hub and preprocessed using QIIME v1.91 scripts (6) provided by the MicrobiomeHelper v2.3 environment (7). Briefly, paired-end sequences were demultiplexed by barcode sequence and split by sample name using default parameters of extract_barcodes, split_libraries_fastq, and split_sequence_file_on_sample_ids scripts. The resulting fastq sequences were exported to R statistical environment v3.5.1 (8) for quality filtering, merging paired reads, and performing taxonomic inference using the DADA2 package following its online pipeline tutorial v1.12 (9) and employing SILVA v132 as the rRNA reference database (10). The resulting data were used to build a phyloseq object (11) for taxonomic abundance representation and diversity estimation. The total number of high-quality reads in the phyloseq object used for characterizing the microbiota ranged between 11,234 and 105,551, with a mean of 34,601 and median of 28,110 reads. The phyloseq object was rarefied using the minimum depth value of 11,200 reads to normalize all samples to the same depth.

**FIG 1:**
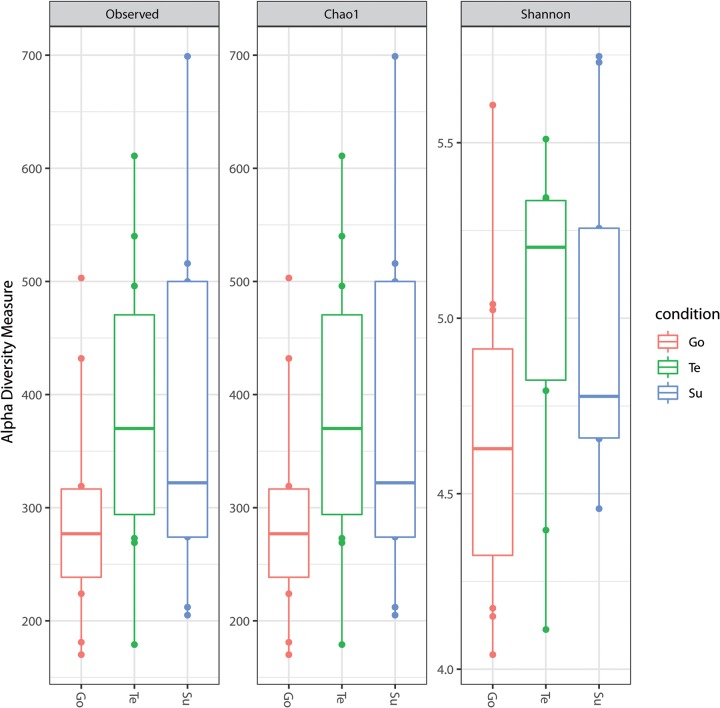
Observed counts and alpha diversity measured by the Chao1 and Shannon indexes in the gonad (Go), surface (Su), and testa (Te).

Based on the amplicon sequence variant (ASV) obtained from DADA2, the main taxonomic classifications at the phylum level were *Proteobacteria*, followed by *Kiritimatiellaeota*, *Fusobacteria*, *Epsilonbacteraeota*, *Cyanobacteria*, and *Bacteroidetes*. On average, the relative abundance of phyla in surface, testa, and gonads was variable (Table 1). Additionally, the observed counts and alpha diversity measured by the Chao1 and Shannon indexes showed that the testa harbors greater taxonomic diversity than the surface and gonads (Fig. 1). This is the first report that shows the microbially diverse populations of Loxechinus albus grown in a controlled culture environment in the Southern Hemisphere.

**TABLE 1:** Relative abundance of the top six phyla represented by 16S rRNA gene amplicon-based bacterial diversity in gonad, surface, and testa

Phylum	Relative abundance (%) of:		
	Gonad	Surface	Testa
*Proteobacteria*	85.4	51.8	70.7
*Epsilonbacteraeota*	4.1	33.6	12.3
*Fusobacteria*	5.3	9.2	6.8
*Bacteroidetes*	4.6	4.9	8.4
*Cyanobacteria*	0.1	0.2	1.0
*Kiritimatiellaeota*	0.5	0.3	0.8

### Data availability.

The raw data and 16S rRNA denoised amplicon sequences were deposited at the ENA EMBL database under the accession number PRJEB33428.
